# Evaluating Meaningful Patient and Public Involvement in Intensive Care Research—A PEIRS‐22 Survey

**DOI:** 10.1111/nicc.70712

**Published:** 2026-10-07

**Authors:** Marie O. Collet, Camilla R. L. Bruun, Anders Granholm, Eva Laerkner, Anne C. Brøchner, Annette L. B. Sørensen, Lone M. Poulsen, Bodil S. Rasmussen, Thomas Strøm, Anne‐Marie G. Bunzel, Camilla B. Mortensen, Louise S. Herløv, Karin Piil, Maj‐Brit N. Kjær

**Affiliations:** ^1^ Department of Intensive Care Copenhagen University Hospital, Rigshospitalet Copenhagen Denmark; ^2^ Department of Clinical Medicine University of Copenhagen Copenhagen Denmark; ^3^ Department of Public Health, Section of Biostatistics University of Copenhagen Copenhagen Denmark; ^4^ Department of Anaesthesiology and Intensive Care Odense University Hospital Odense Denmark; ^5^ Department of Anaesthesiology and Intensive Care Kolding Hospital, University Hospital of Southern Denmark Denmark; ^6^ Department of Regional Health Research University of Southern Denmark Odense Denmark; ^7^ Department of Anaesthesia and Intensive Care Bispebjerg and Frederiksberg Hospital Copenhagen Denmark; ^8^ Department of Anaesthesiology Zealand University Hospital Koege Denmark; ^9^ Department of Anaesthesia and Intensive Care Aalborg University Hospital Aalborg Denmark; ^10^ Department of Clinical Medicine Aalborg University Aalborg Denmark; ^11^ Department of Intensive Care Zealand University Hospital Koege Denmark; ^12^ School of Nursing, Faculty of Health Sciences Curtin University Perth Western Australia Australia; ^13^ Department of Oncology, Centre for Cancer and Organ Diseases Copenhagen University Hospital, Rigshospitalet Copenhagen Denmark

**Keywords:** critical illness, cross‐sectional study, engagement, intensive care, patient and public involvement

## Abstract

**Background:**

Patient and public involvement (PPI) is increasingly recommended in health research to enhance relevance, quality and implementation of findings. In intensive care research, involving patients, family members and healthcare professionals may help ensure that research addresses outcomes that are meaningful to those affected by critical illness.

**Aim:**

This study aimed to assess the level of meaningful PPI engagement during the development of a core outcome set (COS) for general intensive care unit (ICU) patients in Denmark.

**Study Design:**

A cross‐sectional survey using the Danish version of the Patient Engagement in Research Scale (PEIRS‐22) was conducted between December 2024 and January 2025. We invited all members (patients, family, healthcare professionals and researchers) of five research panels, established in 2021 to support COS development across Danish university hospitals (*N* = 46). PEIRS‐22 total scores range from 1 to 100, with higher scores indicating greater meaningful engagement (scores ≥ 70.1 were considered meaningful). Descriptive statistics, item‐level analyses and non‐parametric comparisons between participant roles were performed.

**Results:**

Thirty‐seven of 46 participants responded (80.4%): 27.0% (*n* = 10) were patients/family, 46.0% (*n* = 17) healthcare professionals and 27.0% (*n* = 10) researchers. The median PEIRS‐22 total score was 78 (IQR 68–93), with 72.0% (*n* = 27) scoring ≥ 70.1. Engagement differed statistically significant by role (*p* = 0.004), with researchers reporting the highest scores (median 97), followed by patients/family (89), while healthcare professionals reported lower scores (74).

**Conclusion:**

PPI in COS development for general ICU patients was perceived as meaningful overall. However, differences across stakeholder groups and lower achievement of advanced engagement elements suggest opportunities to strengthen shared decision‐making, role clarity and perceived relevance, particularly among healthcare professionals. Meaningful involvement of patients, families and clinicians may support the development of more patient‐centred intensive care research and outcomes that better reflect the priorities of those affected by critical illness.

**Relevance to Clinical Practice:**

Meaningful PPI can help ensure that intensive care research reflects the priorities of patients and families. Clear roles, clinical relevance, and opportunities to influence decisions may strengthen engagement, particularly among healthcare professionals. Critical care nurses may play an important role in facilitating collaboration between patients, families, clinicians, and researchers.

## Impact Statements

- What is known about the topic
  ○ Patient and public involvement (PPI) is increasingly recommended to improve the relevance, quality and implementation of health research.
  ○ Meaningful engagement requires more than participation alone and should include opportunities for influence, collaboration and shared decision‐making.
  ○ Evidence regarding the quality and meaningfulness of PPI in intensive care research remains limited.
- What this paper adds
  ○ PPI in the development of a core outcome set for general ICU patients was perceived as meaningful by most participants.
  ○ Researchers reported higher levels of meaningful engagement than healthcare professionals, highlighting differences between stakeholder groups.
  ○ Foundational elements of engagement were well established, whereas opportunities for shared decision‐making and co‐production were less consistently achieved.
  ○ Clear roles, clinical relevance and structured collaboration may strengthen meaningful engagement in future intensive care research.

## 1 Introduction

Patient and public involvement (PPI) in health research is increasingly recommended internationally due to its potential to improve the quality, relevance and applicability of research outcomes in clinical practice [1]. The United Kingdom National Institute of Health Research defines PPI as research conducted *with* or *by* patients and the public, rather than *about* or *for* them [2]. Patients' lived experiences provide unique insights, and their involvement reflects a democratic right to participate in research that informs healthcare practice. Consequently, PPI is increasingly encouraged by patient organisations, funding bodies, academic journals and advocacy groups [3].

Evidence suggests that meaningful PPI can improve study relevance, design, recruitment and dissemination [4]. However, PPI also faces important challenges such as tokenism, where patients are involved primarily to fulfil formal requirements rather than to influence decision‐making, and symbolic inclusion, where diversity is present but engagement remains superficial [5]. To be effective, PPI must therefore be respectful, meaningful and integrated throughout the whole or part of the research process.

Traditionally, PPI primarily focused on patients, often overlooking the perspectives of families and informal caregivers [6]. Involvement of both patients and family requires research environments that acknowledge and value their distinct experiences and roles. Moreover, PPI is most commonly implemented during early phases of research, although evidence suggests that sustained involvement across the research stages might enhance its impact [7]. Such involvement can be time‐consuming and challenging, particularly when patients prioritise recovery or caregiving responsibilities, and conflicts may arise due to differing expectations and perspectives between patients and researchers [8].

In Denmark, the implementation and evaluation of PPI is still evolving, particularly within intensive care and cancer research. Common approaches include involving patients as members of advisory boards or research partners [9]. Addressing existing challenges requires clear strategies for integrating PPI throughout the research cycle, as well as systematic evaluation and transparent reporting of its impact [4]. Understanding patients' motivations for participation may further support the design of meaningful and sustainable involvement initiatives [10].

Quantitative evidence on the impact of PPI has been limited, highlighting the need for validated measures to assess the levels of engagement. A systematic review identified 58 tools for measuring health–patient partnership outcomes. However, few instruments assessed both patient and family involvement [11]. The Patient Engagement in Research Scale (PEIRS), which was shortened to 22 items (PEIRS‐22) in 2021 and translated to Danish in 2023, was developed to measure the extent of meaningful patient and family engagement in health research [12, 13, 14].

Critical illness is frequently associated with profound physical, cognitive and psychological consequences that extend beyond hospital discharge and affect both patients and their families [15, 16]. As a result, outcomes considered important by clinicians and researchers may not fully reflect the priorities and experiences of patients and family members. PPI may therefore be particularly valuable in intensive care research to ensure that research addresses outcomes that are meaningful to those directly affected by critical illness.

Within intensive care unit (ICU) research, PPI is increasingly recognised but remains relatively uncommon [17]. However, the development of a core outcome set (COS) for general ICU patients required comprehensive stakeholder involvement to ensure that future research outcomes were relevant and meaningful [18, 19]. In this context, it was important to include not only patients and family but also healthcare professionals with ICU experience and researchers in order to capture diverse perspectives across the research continuum [18]. This study aimed to evaluate the level of meaningful engagement among patients, family members, HCPs and researchers involved in the development of a COS for general ICU patients using the Patient Engagement in Research Scale (PEIRS‐22).

## 2 Methods

### 2.1 Study Design

To evaluate the level of meaningful engagement in the development of the COS, we conducted a cross‐sectional survey using the Danish version of PEIRS‐22 [14]. The survey was distributed in Denmark from December 2024 to January 2025. Data collection comprised a cover letter describing the study purpose, a questionnaire capturing demographic characteristics, and the 22‐item PEIRS‐22 instrument. This study was conducted according to an internally developed study protocol and reported with considerations of the Guidance for Reporting Involvement of Patients and the Public Checklist (GRIPP2) (short version) (Table S1) [20].

### 2.2 Participants

All members who had participated in one of the five research panels involved in developing the COS for the general adult ICU patient were eligible for inclusion [19].

### 2.3 Development and Organisation of the PPI Research Panels

Five PPI research panels were established across five university hospitals in Denmark in autumn 2021 to provide ongoing stakeholder involvement throughout the COS development process. The panels included 10 patients and five family members with lived ICU experience, 23 healthcare professionals (HCPs) with ICU experience including nurses and nine researchers working in or with knowledge of the ICU setting. Patients and family members were recruited through ICU outpatient clinics or local research units, including previous research participants, while HCPs and researchers were recruited through participating ICU departments and research units. The panels met face to face four to five times annually since their establishment. Meetings were used to discuss different stages of the COS development, including methodology, data collection and analysis, emerging findings and dissemination plans. Panel members contributed to specific study activities, including the development of key study materials, including a Delphi survey questionnaire and interview guides. Participation was voluntary and unpaid, and refreshments were provided during in‐person meetings. In addition, one online meeting was conducted with all members to discuss issues relevant to ICU research.

### 2.4 Survey Construct

The survey included the Danish version of PEIRS‐22, consisting of 22 items across seven domains: 1. Procedural requirements (PR), 2. convenience (CN), 3. Contributions (CT), 4. Team environment and interaction (T), 5. Support (SU), 6. Feeling valued (FV) and 7. Benefits (BE) [14]. Items are rated on a 5‐point Likert scale and scored from 0 to 4, with higher scores indicating greater perceived meaningful engagement [12]. The PEIRS‐22 has previously been validated and culturally adapted into Danish [14]. Therefore, no additional pilot testing was conducted prior to survey distribution. The survey package included a cover letter describing the study purpose, demographic questions and the PEIRS‐22 questionnaire (Supporting Information S2).

### 2.5 Survey Administration

The survey was administered electronically using REDCap hosted by Copenhagen University Hospital—Righospitalet. Participants received a pre‐notification email 2 weeks prior to survey distribution. The survey invitation was subsequently sent by email, followed by up to two reminder emails at 2‐week intervals to non‐respondents within the study period.

Participants in this study provided demographic information including age, gender, role (patient, family, HCP or researcher), city of the research panel and duration of participation in the research panels (months).

### 2.6 Statistical Analysis

Descriptive statistics were used to summarise participant characteristics and PEIRS‐22 responses. Categorical variables are presented as counts and percentages, and continuous variables as medians with interquartile ranges (IQR), as several variables were non‐normally distributed.

Only full questionnaires were included in the analysis. One respondent had a single missing item (item T5). There was trust among the research project team members. This questionnaire was retained, and no imputation or correction was performed.

The PEIRS‐22 total score was calculated by summing all 22 item scores (0–4 per item), dividing the raw sum by 88 and multiplying by 100 to yield a percentage score, with higher values indicating more meaningful engagement. Total scores were categorised using established thresholds as low to moderately meaningful (≤ 70.0), moderately meaningful (70.1 to < 82.7), very meaningful (82.7 to < 92.0) or extremely meaningful (≥ 92.0) [14]. For each separate item, a dichotomous indicator was created to reflect whether the respondent scored above the cut‐off for low meaningfulness (score ≥ 3). At the item level, the proportion of participants scoring above this cut‐off was calculated and expressed as a percentage. An item was considered achieved if at least 80% of participants scored above the cut‐off for low meaningfulness [21].

Item‐level analyses were conducted to examine the patterns of meaningful engagement across different levels of engagement complexity. As per the PEIRS‐22 framework, items were categorised into bronze (items CN4, T4, PR14, CT2, BE1, CT1), which was easiest to achieve, silver (items PR2, PR12, PR9, BE2, FV1, T5, SU2, BE4) as intermediate and gold (items FV3, T2, PR13, PR11, CN1, PR10, SU1, CN3) as most difficult to achieve, levels of meaningful engagement. The extent to which engagement was achieved at bronze, silver and gold levels was interpreted descriptively based on the proportion of items meeting this criterion within each category [12].

Comparison of overall PEIRS‐22 total scores across participant roles (patient/family, HCPs and researchers) was performed using non‐parametric tests due to non‐normal score distributions. Patients and family members were analysed as a single group due to low participation in each group and to reduce the risk of identification of individual respondents in the survey data. Given the small subgroup sizes, comparisons across participant roles were considered exploratory and should be interpreted cautiously, with the Kruskal–Wallis test, with post hoc pairwise comparisons performed where appropriate.

Internal consistency of the PEIRS‐22 in the present sample was assessed using Cronbach's alpha. This analysis was performed to describe internal consistency in the study population and was not intended as a validation of the instrument.

All statistical analyses were conducted using SAS Enterprise Guide (version 8.3; SAS Institute Inc., Cary, NC, USA). Two‐sided *p*‐values < 0.05 were considered statistically significant.

### 2.7 Ethical Considerations

The study was conducted in accordance with the General Data Protection Regulation (GDPR). The survey invitation included a cover letter describing data handling, participants' rights, including voluntary participation and that questionnaire submission constituted informed consent for participation of aggregated non‐identifiable results. Participants could exit the survey at any time before submission. Responses were collected and stored without direct identifiers and were treated confidentially. All participants had previously provided verbal consent to participate in the research panels, and informed consent for this survey was reaffirmed through survey completion.

The study was registered with and approved by the Danish Data Protection Agency (P‐2021‐307) date 10.01.2024, as required by national regulations. In accordance with Danish law, ethical committee approval was not required for this type of survey‐based research.

## 3 Results

Of the 46 research panel members invited, 37 completed the survey (80.4%) and were included in the analysis. The median age of the participants was 48 (IQR 44.0–56.0), with 70.3% identified as women. Of those who answered, 27.0% were patients or family, 46.0% were HCPs and 27.0% were researchers (Table 1).

**TABLE 1:** Participants' characteristics.

	All *N* = 37	Patient/family *n* = 10	HCPs *n* = 17	Researchers *n* = 10
Age, *median (IQR)*	48 (44–56)	57 (54–69)	46 (43–50)	48 (46–52)
Sex, *n (%)*				
Women	26 (70.3)	4 (40.0)	14 (82.4)	8 (80.0)
Men	11 (29.7)	6 (60.0)	3 (17.7)	2 (20.0)
Highest completed education, *n (%)*				
Basic school	—	—	—	—
High school or short‐cycle tertiary	6 (16.2)	6 (60.0)	—	—
Higher education	31 (83.8)	4 (40.0)	17 (100.0)	10 (100.0)
Employment status, *n (%)*				
Working (full or part‐time)	32 (86.5)	5 (50.0)	17 (100.0)	10 (100.0)
Not working	5 (13.5)	5 (50.0)	—	—
Relationship status, *n (%)*				
Married/living together	31 (83.8)	6 (60.0)	15 (88.2)	10 (100.0)
Single	6 (16.2)	4 (40.0)	2 (11:8)	—
Month of involvement in the research panels, *n (%)*				
< 12 months	8 (21.7)	3 (30.0)	4 (23.5)	1 (10.0)
≥ 12 month	29 (78.4)	7 (70.0)	13 (76.5)	9 (90.0)
Month of involvement in the research panels, *Median (IQR)*	3 (1–5)	3 (1–5)	3 (2–5)	2 (1–4)

Abbreviations: HCPs, Healthcare professionals; IQR, Interquartile range.

### 3.1 PEIRS‐22 Total Score

Overall, the median PEIRS‐22 total score was 78 (IQR 68.0–93.0), with 72.0% of participants scoring above the meaningfulness threshold of 70.1 (Table 2). Scores differed statistically significant by role (*p* = 0.004); patient and family had a median score of 89 (IQR 65.9–93.2), corresponding to very meaningful engagement; healthcare professionals had a median score of 74 (IQR 64.8–78.4), corresponding to moderately meaningful engagement; and researchers had a median score of 97 (IQR 84.1–100.0), corresponding to extremely meaningful engagement (Table 2). Researchers scored higher than healthcare professionals (*p* = 0.0034), while scores of patients and family did not differ statistically significant from other groups (both *p* ≥ 0.17). Most HCPs (88.2%) rated engagement at or below the level of moderately meaningful. Levels of perceived meaningful engagement also differed across research panel membership (*p* = 0.008). The PEIRS‐22 demonstrated very high internal consistency (Cronbach's alpha = 0.96).

**TABLE 2:** Total scores and subgroups of PEIRS‐22 total score.

	PEIRS‐22 total score median (IQR)	Min–max of total scores	*p*‐Value of difference between groups	% of PPI members above low meaningfulness threshold
All (*n* = 37)	78.0 (68.0–93.0)	55.0–100.0		72.0
Sex			0.378^a^	
Women	75.6 (65.9–92.0)	56.8–100.0		73.1
Men	84.1 (68.2–96.6)	54.5–100.0		72.7
Month of involvement in the research panels			0.001^a^	
< 12 months	64.8 (59.7–70.5)	54.5–78.4		37.5
≥ 12 months	84.1 (75.0–94.3)	94.3–58.0		82.8
Roles			0.004^a^	
Patients/family	88.6 (65.9–93.2)	54.5–100.0		60.0
HCPs	73.9 (64.8–78.4)	58.8–92.0		64.7
Researcher	97.2 (84.1–100.0)	70.5–100.0		80.0

Abbreviation: HCPs, Healthcare professionals.

^a^ Test for subgroup differences in PEIRS‐22 total score was assessed using Kruskal–Wallis tests.

### 3.2 PEIRS‐22 Subdomains

Subdomain scores for the entire group are presented in Table 3. The median scores for procedural requirements, convenience and contributions were above the threshold for meaningful engagement (Table 3). Kruskal–Wallis tests showed statistically significant differences across roles for domains of procedural requirements, convenience, support, feeling valued and benefits (all *p* < 0.03). Contributions and team environment did not differ statistically significant (Table 3).

**TABLE 3:** Scores across subdomains for all groups.

Subdomain	Cut‐point score for low meaningfulness^a^	Median (IQR)	*p*‐Value^b^ between groups	Min‐Max	% of PPIs above low meaningfulness threshold
PR	22.3	23.0 (20.0–28.0)	0.007	12–28	54.1
CN	9.6	9.0 (7.0–11.0)	0.002	4–12	45.9
CT	9.6	11.0 (9.0–12.0)	0.076	5–12	56.8
T^c^	6.4	6.0 (6.0–8.0)	0.107	3–8	43.2
SU	6.4	6.0 (5.0–8.0)	0.025	4–8	37.8
FV	6.4	6.0 (5.0–7.0)	0.019	4–8	40.5
BE	9.6	9.0 (7.0–11.0)	0.004	5–11	43.2

Abbreviations: BE, benefits; CN, convenience; CT, contributions; FV, feel valued; PR, procedural requirement; SU, support; T, team environment.

^a^ Defined by Hamilton et al. (2021) [12].

^b^ Difference between groups assessed by the Kruskal–Wallis test.

^c^ One missing in this item; no imputation was done (analyses are conducted using complete cases only).

### 3.3 Bronze, Silver and Gold Levels

We examined the proportion of participants endorsing a positive experience for each PEIRS‐22 item, with levels of meaningful engagement and areas of lower engagement presented in Table 4. All bronze‐level items met the criterion of 80% achievement (100%), compared with 62.5% of silver‐level items and 25% of gold‐level items. This suggests that PPI performed well on elements that are easiest to achieve (bronze), less consistently in intermediate elements (silver) and least on advanced elements (gold), indicating room for improvement, particularly in intermediate and advanced engagement. Nine items had a score < 80.0%. Three items had only < 70.0% of PPI reporting meaningful engagement; item CN1*: I had the opportunity to provide input into selecting my tasks for the project*, item BE4: *My involvement had a positive impact on my life*, and item CN3: *Throughout the project, I had sufficient time to complete my tasks for the project*. Other items that were close to the cut‐off, with 75.7%–78.4% of respondents affirming positive experiences, were PR11: *I participated in making decisions about the project*, SU2: *Any concerns I had were addressed* and PR13: *Communication within the research team was clear throughout the project*.

**TABLE 4:** Participants' degrees of meaningful engagement and areas of low meaningful engagement captured using PEIRS‐22.

Participant	Total PEIRS22 score	Procedural requirements	Convenience	Contributions	Team environment & interaction	Support	Feel valued	Benefits
PT/FA	94.32	27	11	11	8	8	7	11
PT/FA	65.91	17	8	11	6	4	5	7
PT/FA	62.50	17	7	8	6	4	5	8
PT/FA	84.09	28	10	9	6	5	6	10
PT/FA	93,18	25	10	12	8	8	8	11
PT/FA	100.00	28	12	12	8	8	8	12
PT/FA	93.18	25	11	11	8	8	7	12
PT/FA	68.18	20	8	9	3	6	6	8
PT/FA	54.55	15	4	8	5	4	5	7
PT/FA	93.18	26	12	11	8	6	7	12
HCPs	92.05	28	10	12	8	7	6	10
HCPs	76.14	21	8	9	8	4	7	10
HCPs	73.86	21	9	9	6	6	6	8
HCPs	71.59	22	6	12	3	5	6	9
HCPs	78.41	21	6	12	6	8	7	9
HCPs	59.09	18	6	9	5	4	4	6
HCPs	63.64	18	6	9	6	6	6	5
HCPs	80.68	27	12	9	6	6	5	6
HCPs	87.50	23	11	11	8	7	8	9
HCPs	65.91	18	7	10	5	5	5	8
HCPs	70.45	19	8	12	5	5	5	8
HCPs	78.41	24	7	11	8	5	6	8
HCPs	75.00	23	9	9	6	6	5	8
HCPs	64.77	20	7	8	6	4	6	6
HCPs	76.14	23	8	9	7	6	6	8
HCPs	56.82	12	5	9	6	5	6	7
HCPs	57.95	20	5	5	6	5	5	5
Researcher	100.00	28	12	12	8	8	8	12
Researcher	96.59	28	11	11	8	8	8	11
Researcher	100.00	28	12	12	8	8	8	12
Researcher	100.00	28	12	12	8	8	8	12
Researcher	97.73	28	10	12	8	8	8	12
Researcher	100.00	28	12	12	8	8	8	12
Researcher	84.09	25	9	11	6	6	7	10
Researcher	75.00	22	12	9	6	4	6	7
Researcher	92.05	28	12	12	6	8	5	10
Researcher	70.45	20	8	9	6	6	6	7

*Note:* PT/FA = Patients/Family, HCPs = Healthcare Professionals. The degree of meaningfulness for the total PEIRS‐22 score heatmap: 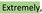, 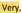, 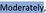, 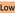. For subdomains, only the low level of meaningfulness is indicated.

## 4 Discussion

In this study evaluating PPI in the development of a COS for the general adult ICU patient, overall engagement was perceived as meaningful. The median PEIRS‐22 total score was 78, with 72% scoring above the predefined threshold. Engagement differed statistically significant across stakeholder roles, with researchers reporting the highest levels, followed by patients and family, while HCPs reported lower levels. Subdomain analyses indicated strengths in procedural requirements, convenience and contributions, whereas more advanced aspects, such as decision‐making influence, time allocation and personal benefit, were less consistently achieved. The bronze–silver–gold analysis demonstrated that foundational elements were well established, while intermediate and advanced levels of engagement showed room for improvement. The relatively high level of meaningful engagement observed across stakeholder groups is particularly important in intensive care research, where patients and family members are recognised as key stakeholders in defining outcomes that reflect recovery, quality of life and the long‐term consequences of critical illness. Meaningful involvement may, therefore, contribute not only to the quality of the research process but also to the relevance and acceptability of future research outcomes.

Several elements supported meaningful engagement. Structural and procedural aspects were strong, with high scores in the ‘Procedural requirements’ and ‘Convenience’ indicating that meetings were accessible and manageable. Consistent achievement of all bronze‐level items suggests that core components, such as being invited, included and able to contribute, were successfully implemented. Participants across roles reported similar scores for ‘Contributions’ and ‘Team Environment’, indicating a collaborative atmosphere that enabled sharing of perspectives. This may reflect the regular meeting structure, diverse stakeholder inclusion and the involvement in concrete tasks such as developing the Delphi survey and interview guide. These findings suggest that clarity of purpose, continuity and tangible tasks are key enablers of meaningful PPI, consistent with previous research [22].

Exploratory comparisons found statistically significant lower engagement among HCPs than researchers. For HCP, meaningfulness may be closely linked to clinical relevance and applicability in daily practice [23]. If the connection between COS methodology and patient care is unclear, engagement may be perceived as abstract or less rewarding [24]. In contrast, researchers may experience stronger alignment between PPI and their professional identity, contributing to higher engagement [25]. HCPs may also feel cautious in discussions with patients and family, potentially limiting openness and contribution [26]. Additionally, the perception of ‘giving more than receiving’ may influence engagement, particularly if time investment is not balanced by perceived benefit. This aligns with previous research highlighting how power dynamics and tokenistic PPI may limit reciprocal value [27]. These findings underscore the importance of clear communication of clinical relevance and expectations for HCPs. The lower engagement reported by HCPs may be particularly important in intensive care research, as clinicians often play a key role in translating research findings into practice. If HCPs do not perceive involvement activities as meaningful or relevant, opportunities to support implementation and uptake of research findings may be reduced.

Although foundational engagement was strong, fewer participants reported positive experiences at silver and gold levels. Items related to decision‐making, time, task selection and personal benefit were below the 80% threshold. This suggests that, while participants were included, deeper shared ownership and empowerment were less consistently achieved. Opportunity to influence tasks and decision‐making appears central to advancing towards co‐production [28]. Ensuring adequate time and addressing concerns may further strengthen engagement, as repeated interactions and supportive group processes have been shown to foster trust, confidence and more active participation [29].

These findings have practical implications for our ongoing COS programme and future PPI in ICU research. Meaningful engagement requires clear structures, accessible processes and defined roles [30]. For HCPs, engagement may be strengthened by clarifying clinical relevance, roles and expectations. For patients and family members, greater opportunities to select tasks, influence decisions and raise concerns may support deeper involvement. Early clarification of roles and expectations may reduce uncertainty, particularly for participants with dual roles [31], while shared decision‐making is important for moving beyond consultation [30]. Meeting structure will allow sufficient time for contributions and explicitly invite concerns and feedback. Critical care nurses may be particularly well positioned to facilitate psychologically safe dialogue and collaboration between patients, families, clinicians and researchers. Rather than using PEIRS‐22 solely as an end‐point evaluation, repeated assessments could identify areas of less meaningful engagement during the research process and guide adjustments to panel activities. Combining PEIRS‐22 assessments with qualitative discussion may further clarify why particular aspects of engagement are less meaningful and how they can be improved.

### 4.1 Strength and Limitations

A key strength of this study is the use of a validated instrument (PEIRS‐22) with high internal consistency and inclusion of diverse stakeholder groups across multiple university hospitals. The relatively high response rate (80%) strengthens internal validity. However, the relatively small sample size for statistical comparisons may have limited the ability to detect differences between groups (risk of type II error). Although the study reflects substantial stakeholder involvement, subgroup sizes were small; therefore, subgroup comparisons should be considered exploratory. The participant sample was younger (mean age < 50 years) and predominantly women, in contrast to the general ICU population, which is typically older than 65 years and predominantly men. These differences in demographic characteristics may have influenced the findings. Additionally, engagement was assessed through self‐report at a single time point, which may be subject to recall and social desirability bias, and the absence of qualitative data limited exploration of why engagement differed across participants. Finally, the findings may have limited generalisability beyond similar ICU research settings and healthcare contexts.

### 4.2 Implications for Clinical Practice

Structured and ongoing PPI, supported by clear roles and shared decision‐making, may help align ICU research with patient and family priorities, while critical care nurses may facilitate collaboration between patients, families and researchers.

## 5 Conclusion

PPI in the development of a COS for the general ICU patients was overall perceived as meaningful, particularly at foundational levels. However, differences across stakeholder roles and lower scores in advanced engagement domains indicate opportunities to strengthen shared decision‐making, clarify relevance and enhance perceived value, particularly for HCP. Future PPI initiatives in ICU research should build on strong structural foundations while intentionally advancing towards deeper and more reciprocal forms of engagement.

## Funding

This work was supported by the Dagmar Marshall Foundation.

## Ethics Statement

The study ‘Evaluation of engagement in Patient and Public Involvement in developing a core outcome set – a PEIRS‐22 survey’ was conducted in accordance with the Declaration of Helsinki and relevant national regulations, including the General Data Protection Regulation (GDPR).

Participants received written information outlining the study purpose, data handling procedures and their rights. Participation was voluntary, and completion and submission of the questionnaire were considered to imply informed consent. Participants were able to withdraw from the survey at any time prior to submission. Data were collected and stored without direct identifiers and handled confidentially.

All participants had previously provided verbal consent to participate in the research panels, and informed consent for the present survey was reaffirmed through questionnaire completion. The study was registered with and approved by the Danish Data Protection Agency (P‐2021‐307). In accordance with Danish legislation, ethical committee approval was not required for this type of survey‐based study.

## Conflicts of Interest

The authors declare that they have no known competing financial interests or personal relationships that could have appeared to influence the work reported in this paper.

The authors declare the following financial interests/personal relationships which may be considered as potential competing interests: Marie Oxenboell Collet reports that financial support was provided by Novo Nordisk Foundation and Dagmar Marshal Foundation.

## Supporting information


**Table S1:** GRIPP2 short form.
**Supporting Information:** S2. Survey.

## Data Availability

The anonymised data set generated during the current study is available from the corresponding author upon reasonable request and in accordance with the General Data Protection Regulation (GDPR).
