# Supplementary material for: Evaluating Meaningful Patient and Public Involvement in Intensive Care Research—A PEIRS‐22 Survey
Source: Nurs Crit Care. 2026 Oct 7;31(6):e70712. doi: 10.1111/nicc.70712 (PMC13646686; doi:10.1111/nicc.70712)
Supplement: Supplementary file 1 [file NICC-31-0-s001.docx]

**Supplementary material (SM)**

**Evaluation of engagement in Patient and Public Involvement in developing a core outcome set – a PEIRS-22 survey**

**Table of Contents**

[Table SM1. GRIPP2 short form.	2](#_Toc239833255)

[SM2. Survey.	3](#_Toc239833256)

# **Table SM1.** GRIPP2 short form.

| Section and topic | Item | Reported on page No |
| --- | --- | --- |
| 1: Aim | Report the aim of PPI in the study | 3, 4, 5 |
| 2: Methods | Provide a clear description of the methods used for PPI in the study | 4,5 |
| 3: Study results | Outcomes—Report the results of PPI in the study, including both positive and negative outcomes | 7 |
| 4: Discussion and conclusions | Outcomes—Comment on the extent to which PPI influenced the study overall. Describe positive and negative effects | 8,9 |
| 5: Reflections/critical perspective | Comment critically on the study, reflecting on the things that went well and those that did not, so others can learn from this experience | 8,9 |

PPI=patient and public involvement

Staniszewska S, Brett J, Simera I, Seers K, Mockford C, Goodlad S, Altman DG, Moher D, Barber R, Denegri S, Entwistle A, Littlejohns P, Morris C, Suleman R, Thomas V, Tysall C. *GRIPP2 reporting checklists: tools to improve reporting of patient and public involvement in research.* Res Involv Engagem. 2017 Aug 2;3:13. doi: 10.1186/s40900-017-0062-2. PMID: 29062538; PMCID: PMC5611595.

# **SM2.** Survey.

**Patient Engagement In Research Scale – PEIRS-22**

| Name: | |  | |
| --- | --- | --- | --- |
| Date: | |  | |
| Your project’s name: | | |  |
|  |  | |  |

**INSTRUCTIONS:** Thinking about your experience as a patient partner in the project, please respond to the statements by choosing only one box for each statement. If you are unsure about which option to choose for a statement, please give the best response you can. This questionnaire may take you about 3 to 7 minutes to complete.

**Procedural Requirements**

The following seven (7) statements are about your general experiences throughout the project.

| PR2. | The research team members were properly introduced to each other | | | | | |
| --- | --- | --- | --- | --- | --- | --- |
|  | | Strongly Agree  **☐** | Agree  **☐** | Neutral  **☐** | Disagree  **☐** | Strongly Disagree  **☐** |

| PR9. | In general, I had sufficient opportunities to contribute to the project | | | | | |
| --- | --- | --- | --- | --- | --- | --- |
|  | | Strongly Agree  **☐** | Agree  **☐** | Neutral  **☐** | Disagree  **☐** | Strongly Disagree  **☐** |

| PR10. | I was able to perform my tasks for the project | | | | | |
| --- | --- | --- | --- | --- | --- | --- |
|  | | Strongly Agree  **☐** | Agree  **☐** | Neutral  **☐** | Disagree  **☐** | Strongly Disagree  **☐** |

| PR11. | I participated in making decisions about the project | | | | | |
| --- | --- | --- | --- | --- | --- | --- |
|  | | Strongly Agree  **☐** | Agree  **☐** | Neutral  **☐** | Disagree  **☐** | Strongly Disagree  **☐** |

| PR12. | I received sufficient updates about the project | | | | | |
| --- | --- | --- | --- | --- | --- | --- |
|  | | Strongly Agree  **☐** | Agree  **☐** | Neutral  **☐** | Disagree  **☐** | Strongly Disagree  **☐** |

| PR13. | Communication within the research team was clear throughout the project | | | | | |
| --- | --- | --- | --- | --- | --- | --- |
|  | | Strongly Agree  **☐** | Agree  **☐** | Neutral  **☐** | Disagree  **☐** | Strongly Disagree  **☐** |

| PR14. | The project was worth the time I spent on it | | | | | |
| --- | --- | --- | --- | --- | --- | --- |
|  | | Strongly Agree  **☐** | Agree  **☐** | Neutral  **☐** | Disagree  **☐** | Strongly Disagree  **☐** |

**Convenience**

The following three (3) statements are about how convenient it was for you to contribute throughout the project.

| CN1. | I had the opportunity to provide input into selecting my tasks for the project | | | | | |
| --- | --- | --- | --- | --- | --- | --- |
|  | | Strongly Agree  **☐** | Agree  **☐** | Neutral  **☐** | Disagree  **☐** | Strongly Disagree  **☐** |

| CN3. | Throughout the project, I had sufficient time to complete my tasks for the project | | | | | |
| --- | --- | --- | --- | --- | --- | --- |
|  | | Strongly Agree  **☐** | Agree  **☐** | Neutral  **☐** | Disagree  **☐** | Strongly Disagree  **☐** |

| CN4. | I had opportunities to express my views | | | | | |
| --- | --- | --- | --- | --- | --- | --- |
|  | | Strongly Agree  **☐** | Agree  **☐** | Neutral  **☐** | Disagree  **☐** | Strongly Disagree  **☐** |

**Contributions**

The following three (3) statements are about your contributions throughout the project.

| CT1. | I contributed by providing my perspective | | | | | |
| --- | --- | --- | --- | --- | --- | --- |
|  | | Strongly Agree  **☐** | Agree  **☐** | Neutral  **☐** | Disagree  **☐** | Strongly Disagree  **☐** |

| CT2. | My contributions were a good use of my time | | | | | |
| --- | --- | --- | --- | --- | --- | --- |
|  | | Strongly Agree  **☐** | Agree  **☐** | Neutral  **☐** | Disagree  **☐** | Strongly Disagree  **☐** |

| CT4. | My workload in the project was manageable | | | | | |
| --- | --- | --- | --- | --- | --- | --- |
|  | | Strongly Agree  **☐** | Agree  **☐** | Neutral  **☐** | Disagree  **☐** | Strongly Disagree  **☐** |

**Team Environment and Interaction**

The following two (2) statements are about the research environment and interaction throughout the project.

| T2. | I was an equal partner in the research project team | | | | | |
| --- | --- | --- | --- | --- | --- | --- |
|  | | Strongly Agree  **☐** | Agree  **☐** | Neutral  **☐** | Disagree  **☐** | Strongly Disagree  **☐** |

| T5. | There was trust among the research project team members | | | | | |
| --- | --- | --- | --- | --- | --- | --- |
|  | | Strongly Agree  **☐** | Agree  **☐** | Neutral  **☐** | Disagree  **☐** | Strongly Disagree  **☐** |

**Support**

The following two (2) statements are about the support provided throughout the project.

| SU1. | I received sufficient support to contribute to the project (for example, orientation, readings, training workshops, webinars) | | | | | |
| --- | --- | --- | --- | --- | --- | --- |
|  | | Strongly Agree  **☐** | Agree  **☐** | Neutral  **☐** | Disagree  **☐** | Strongly Disagree  **☐** |

| SU2. | Any concerns I had were addressed | | | | | |
| --- | --- | --- | --- | --- | --- | --- |
|  | | Strongly Agree  **☐** | Agree  **☐** | Neutral  **☐** | Disagree  **☐** | Strongly Disagree  **☐** |

**Feel Valued**

The following two (2) statements are about your feeling of being a valued member of the research team.

| FV1. | The research project team appreciated my contributions | | | | | |
| --- | --- | --- | --- | --- | --- | --- |
|  | | Strongly Agree  **☐** | Agree  **☐** | Neutral  **☐** | Disagree  **☐** | Strongly Disagree  **☐** |

| FV3. | I was offered sufficient recognition for my contributions (for example, payment, authorship, or gifts) | | | | | |
| --- | --- | --- | --- | --- | --- | --- |
|  | | Strongly Agree  **☐** | Agree  **☐** | Neutral  **☐** | Disagree  **☐** | Strongly Disagree  **☐** |

**Benefits**

The following three (3) statements are about the benefits of your involvement in the project.

| BE1. | I enjoyed being a part of the project | | | | | |
| --- | --- | --- | --- | --- | --- | --- |
|  | | Strongly Agree  **☐** | Agree  **☐** | Neutral  **☐** | Disagree  **☐** | Strongly Disagree  **☐** |

| BE2. | I made an impact on the decisions in the project | | | | | |
| --- | --- | --- | --- | --- | --- | --- |
|  | | Strongly Agree  **☐** | Agree  **☐** | Neutral  **☐** | Disagree  **☐** | Strongly Disagree  **☐** |

| BE4. | My involvement had positive impacts on my life | | | | | |
| --- | --- | --- | --- | --- | --- | --- |
|  | | Strongly Agree  **☐** | Agree  **☐** | Neutral  **☐** | Disagree  **☐** | Strongly Disagree  **☐** |
